# Uranyl Acetate Induces Oxidative Stress and Mitochondrial Membrane Potential Collapse in the Human Dermal Fibroblast Primary Cells

**Published:** 2012

**Authors:** Bahram Daraie, Jalal Pourahmad, Neda Hamidi-Pour, Mir-Jamal Hosseini, Fatemeh Shaki, Masoud Soleimani

**Affiliations:** a *School of Pharmacy, Shaheed Beheshti University of Medical Sciences, Tehran, Iran.*; b *Pharmaceutical Sciences Research Center, Shaheed Beheshti University of Medical Sciences, Tehran, Iran.*; c *Department of Toxicology, Faculty of Medical Sciences, Tarbiat Modares University, Tehran, Iran.*; d *Student Research Committee, School of Pharmacy, Shahid Beheshti University of Medical Sciences, Tehran, Iran. *; e *Department of Hematology, Faculty of Medical Sciences, Tarbiat Modares University, Tehran, Iran.*

**Keywords:** Uranium, Fibroblast, Lipid peroxidation, Oxidative stress

## Abstract

Cytotoxicity of depleted uranium, as a byproduct of military has been came to spotlight in recent decades. DU is known as a chemical rather than radioactive hazard and efforts to illustrating its mechanism is undergo, but the precise complete molecular mechanisms are still unclear. Recent studies showed that uranium induces biological changes in many different target tissues, such as the kidney, brain and skin. The aim of this study was to assess the impact of depleted uranium exposure at the cellular level in the human dermal fibroblast primary cells. The human dermal fibroblast primary cells incubated with different concentration (250-750 μM) of depleted uranium.

Cytotoxicity and mitochondrial function in this cell lines were determined with the LDH leakage assay and the MTT test respectively. MDA levels were measured for determination of Lipid peroxidation in DU treated cells. Besides glutathione depletion and apoptosis phenotype detection were also assessed to complete the mechanistic screening. Results showed that the cell viability ameliorates in concentration and time dependent manners following in 24, 48 and 72 h incubation with DU. Moreover the significant increase in lipid peroxidation and significant decrease in cellular GSH recorded in DU treated human dermal fibroblast primary cells suggesting the preoxidant effect of uranyl ions. Cytoprotective effects of N-acetylcysteine (NAC) and dramatic decrease of cell viability in buthionin sulfoxamid (BSO) pretreated cells indicated the possibility of a critical role for glutathione system in DU detoxification. Death pattern, in fibroblast cells following DU treatment was varied from apoptosis to necrosis while the time and concentration increased.

Since ROS formation is the initiation step for cell apoptosis, the present studies suggest Uranyl-induced toxicity in the human dermal fibroblast primary cells originated from oxidative stress and lead to occurrence of programmed cell death.

## Introduction

Uranium (U) is a ubiquitous environmental trace metallo-element which is found in small amounts in food and water supplies as a non-essential inorganic component (1).The U remaining after removal of the enriched fraction is referred to as depleted U (DU) (2, 3).

It has been documented that inhalation is the most common route of uranium exposure. But uranium can also be absorbed through the skin or wounds, ingested with food or drink, and injected into the system intravenously (4). In recent years, it has been noticed that depleted uranium (DU) in munitions is also another route of exposure to the heavy metal through contact with the skin (5).

The most important toxic mechanism that suggested for uranium is involvement of reactive oxygen species (ROS). ROS are known to play a dual role in biological systems, since they can be either harmful or beneficial to living systems (6). The harmful effects of ROS are balanced by the antioxidant action of non-enzymatic antioxidants in addition to antioxidant enzymes. A number of studies have focused on metal-induced toxicity and carcinogenicity, emphasizing their role in the generation of ROS in biological systems and the significance of this therein (7, 8). 

Previous studies also showed that oral uranyl acetate (UA) administration increased TBARS in kidneys and testes (9). Other studies demonstrated that chronic uranyl nitrate (UN) ingestion resulted in an increase in the levels of free radicals (10), brain lipid peroxidation (LPO) (11) and also uranium induce oxidative stress in lung epitehelial cells and loss of antioxidant response (5).In our previous study, UA caused rapid glutathione oxidation, ROS formation, lipid peroxidation and decreased mitochondrial membrane potential in isolated rat hepatocytes (12). These may be based upon uranium related induction of cellular oxidative stress (13). Taken together with the studies on uranium toxicity it reveals that DU could be an environmental health hazard and requires further investigation to figure out the precise mechanism of its action on its many different target organs. Considering that skin contact is a way of the exposure to uranium, we have investigated the effect of DU on human dermal fibroblast primary cells and to study the response due to induction of oxidative stress.

## Experimental

*Chemicals*

Uranyl acetate and all other chemicals were obtained from the Sigma Chemical Co. with the highest commercial grade available unless otherwise stated.

*Cell cultures and treatments*

The human dermal fibroblast primary cells obtained from National Cell Bank of Iran, Pasteur Institute of Iran (Tehran, Iran) was cultured in DMEM medium supplemented with 10% heat inactivated FBS, 100 IU/mL penicillin, 100 µg/mL streptomycin and 2 mM L-glutamine and maintained in a humidified atmosphere with 5% CO2 at 37°C. The cultured cells were sub-cultured twice each week and the exponentially growing cells were used for all treatments. Experiments were performed in 4 groups of cells as follow: Group Ι: control group, Group П, Ш and IV were pretreated with different dose of uranyl acetate (250-500-750 µM).Only one dose of uranyl acetate was added to each medium.

**Table1:** Mitochondrial function assessment for the human dermal fibroblast primary cells following exposure to uranyl acetate for 24, 48 and 72 h based on the dose-response curves as derived from the MTT assay. Succcinate dehydrogenase activity inhibited by U in a dose- and time-dependent manner (*p < 0.05).

**Treated cells**	**LDH leakage**		
	**24 h**	**48 h**	**72 h**
Control	0 ± 3	3 ± 4	4 ± 6
U (250 µM)	*12 ± 4	34 ± 5*	43 ± 5*
U (500 µM)	23 ± 8*	48 ± 9*	59 ± 8*
U (750 µM)	31 ± 3*	52 ± 6*	82 ± 5*

Data represent the means of three separate experiments (±SD).

*p < 0.05, significantly different when compared with control.

*Lactate dehydrogenase (LDH) leakage assay*

Cytotoxicity induced by uranyl acetate was assayed by LDH leakage into the culture medium. Following exposure to the uranyl acetate the fibroblast cells medium was aspirated and centrifuged at 3000 rpm for 5 min for obtain a cell free supernatant. LDH activity in the medium was measured by specific commer­cial kit from Sigma-Aldrich Co. The viability was measured spectrophotometrically in absorbance at 340 nm. Cell viability was determined relative to the untreated control cells (14).

*Lipid peroxidation*

For this assay human dermal fibroblast cells in exponential growth phase (1×106 cells) were seeded in 75 cm^2^ cell culture flasks. After reaching to 80% confluence, cells were treated with 250, 500, 750 µM uranyl acetate. In the time intervals of 24, 48 and 72 h cells were harvested with a rubber policeman and collected cells were centrifuged at1000 g for 10min at 40°C. The supernatant were removed and cell pellet were suspended in cold PBS and sonicated. 2 mL TBA solution (3.75 g TCA, 92.5 g TBA and 0.7 mL HCl in 100 mL distilled water) were added to 1 mL cell suspension and were heated at 90^o^C for 30 min. Calorimetric absorption was measured at 530 nm (15).

*MTT assay*

Fibroblast cells were suspended in RPMI 1640 at 10^6 ^cells/mL. A sample of 100 µL of the cell suspension was seeded into a 96-well plate. After an overnight culture, cells were exposed to uranyl acetate (0–750 µM) for 24, 48 and 72 h. After incubation with MTT (0.5 mg/mL) for 24 h, the medium was removed and 150 µL of DMSO was added to dissolve the formazon crystals. Absorbance was measured at 540 nm using an ELISA microplate reader. Mitochondrial function was determined relative to the untreated control cells. Data represent the mean value and standard deviation of triplicate assays in at least one experiment (16).

*Intracellular GSH assessment*

GSH content was determined according to the spectrophotometer method for the human dermal fibroblast cells. The content of GSH was determined using DTNB as indicator (17).

*Apoptosis phenotype detection test*

Cells were plated in 96-well plates at a concentration of 5 × 103 cells/well. 24 h after plating, they were washed twice with 200 μL of serum-free medium and were starved by incubation in serum-free medium for 15 h at 37 °C in a 5% CO2 incubator. After starvation, cells were treated with 250, 500, 750 µM uranyl acetate 24, 48 and 72 h. Apoptosis was analyzed using Biovision Annexin-FITC detection kit. At the end of treatment, media from control and uranyl-treated cells were discarded and the cells were washed with 1× phosphate-buffered saline (PBS). Then 200µl buffer solution containing 5 µg/mL annexin-FITC was added to 3 well. In order to detection necrosis, 200 µL buffer solution containing 5 µg/mL PI was added to 3 other wells. After 5 min the supernatant discarded and cells were washed with 1× PBS. 100 µL PBS was added to each well. The fluorescence emission at 530 nm (for annexin v-FITC) and 640 nm (for PI) was measured using a synergy fluorimeter. When calculating the apoptosis and necrotic cell percents it was considered that cells that are apoptotic bind annexin V and necrotic cells with a disrupted plasma membrane display uptake of both annexin V and propidium iodide (18, 19).

*Statistical analysis*

 Levene’s test was used to check the homogeneity of variances. Data were analyzed using one-way analysis of variance (ANOVA) followed by Tukey’s HSD as the post-hoc test. Results were presented as mean ± SD of triplicate samples. The minimal level of significance chosen was p < 0.05.

## Results

The human dermal fibroblast primary cells were exposed to different concentration of uranyl acetate (0-750 µm) for 24, 48 and 72 h and cytotoxicity was determined with the LDH leakage assay. The viability results were shown in Table1.Our results showed the EC_50 _value of 400 µM using the LDH leakage assay when fibroblast cells were exposed to uranyl acetate in 72 h. On the other hand, pretreatment of the human dermal fibroblast primary cells with NAC significantly increased in viability by DU (750 µM) in 24 and 72 h (p < 0.05, Figure 1).

The extent of lipid peroxidation was measured using of thiobarbituric acid reactive substances (TBAR_S_) formation assay (15). The MDA concentration was significantly raised in the human dermal fibroblast primary cells while treated with different concentration of uranyl acetate at different sampling times (24, 48 and 72 h). (p < 0.05; Table 2).

**Table 2:** TBARs level in the human dermal fibroblast primary cells at different concentration and times after uranyl acetate exposure

**Treated cells**	**MDA(µmol/mg protien)**		
	**Incubation time**		
	**24 h**	**48 h**	**72 h**
Control	3.26 ± 0.2	3.21 ± 0.1	3.41 ± 0.1
U (250 µM)	3.59 ± 0.1	5.7 ± 0.17*	12.55 ± 0.38*
U (500 µM)	4.13 ± 0.12	6.9 ± 0.21*	19.33 ± 0.59*
U (750 µM)	4.87 ± 0.15	8.96 ± 0.2*	32.86 ± 1.01*

Data represent the means of three separate experiments (±SD).*p < 0.05, significantly different when compared with control

As shown in Table 3, GSH levels of DU-treated cells were significantly decreased as concentration and time dependent manner (p < 0.05, Table 3) that our result is similar with previous studies. On the other hand, rapid decrease of cell viability in buthionin sulfoxamid (BSO) pretreated cells that depleted cellular content of GSH, indicated the possibility of a critical role for glutathione system in DU detoxification.

**Table 3:** Effect of uranyl acetate on mitochondrial function (suucinate dehydrogenase activity) in the human dermal fibroblast primary cells for 24, 48 and 72 h

**Treated cells**	**MTT assay**		
	**24 h**	**48 h**	**72 h**
Control	0	2 ± 1	3 ± 1
U(250 µM)	9 ± 5	30 ± 7*	40 ± 6*
U(500 µM)	21 ± 3*	43 ± 8*	54 ± 5*
U(750 µM)	27 ± 7*	49± 6*	76 ± 9*
NAC + U(750 µM)	0 ± 6†	46 ± 3	59 ± 9†

Data represent the means of three separate experiments (±SD).

*p < 0.05, significantly different when compared with control.

†p < 0.05, significantly different when compared with U (750 µM)

The MTT dye is metabolized by viable mitochondria to a colored product and can be detected photometric instrument. Thus, the extent ofMTT metabolism is an indicator of mitochondrial function (20). So we used MTT test for assessment of mitochondrial function in fibroblast cells after exposure of different concentration of uranyl acetate. As shown in Table 3, mitochondrial function significantly decreased as concentration and time dependent manner.

**Table 4:** Effect of Uranyl acetate on GSH level in the human dermal fibroblast primary cells at different times

**Treated cells**	**GSH (µmol/mg protien)**		
	**Incubation time**		
	**24 h**	**48 h**	**72 h**
Control	2.01 ± 0.04	2. ± 0.01	2 ± 0.01
U (250 µM)	1.81 ± 0.05*	1.41 ± 0.07*	0.69 ± 0.02*
U (500 µM)	1.7 ± 0.06*	1.16 ± 0.05*	0.44 ± 0.01*
U (750 µM)	1.48 ± 0.05*	0.98 ± 0.03*	0.3 ± 0.01*

Data represent the means of three separate experiments (±SD).

*Significant difference in comparison with control cells (p < 0.05).

 In detection of apoptosis, DU also increased apoptosis signaling in the human dermal fibroblast primary cells following 24, 48 and 72 h after incubation (Table 5). After initiation of apoptosis, phosphatidyl serine gradually appears on the outer leaflet of the plasma membrane of primary cells due to inhibition of Mg-ATP dependent amino phospholipid translocase giving off red fluorescence. The human dermal fibroblast primary cells that bind to Annexin V, which is representative of cells undergoing apoptosis and necrotic cells with a disrupted plasma membrane display uptake of both annexin V and propidium iodide. Du in different concentration showed significant increase in the number of apoptotic phenotype in the human dermal fibroblast primary cells compared to control (Table 5).

**Table 5:** Effect of Uranyl acetate on apoptosis and necrosis phenotype in the human dermal fibroblast primary cells at different times

**Treated cells**	**Incubation time**								
	**24 h**			**48 h**			**72 h**		
	**Cell death%**	**A %**	**N%**	**Cell death%**	**A %**	**N%**	**Cell death%**	**A %**	**N%**
Control	0	0	0	3	97 ± 1	3 ± 1	8	93 ± 3	7 ± 2
U (250 µM)	11	98 ± 5	2 ± 0	30	82± 3*	18 ± 2*	42	74 ± 3*	26 ± 2*
U (500 µM)	20	92 ± 3	8 ± 1*	45	80 ± 1*	20 ± 1*	54	52 ± 2*	48 ± 1*
U (750 µM)	32	89 ± 3	11 ± 3*	48	73 ± 4*	27 ± 3*	72	20 ± 1*	80 ± 1*

A: Apoptosis, N: Necrosis. Data represent the means of three separate experiments (±SD).

*Significant difference in comparison with control cells (p < 0.05).

## Discussion

Oxidative stress, an imbalance between free radical generation and the antioxidant defense system, represents a common threat and danger for all aerobic organisms. ROS can be generated by endogenous physiological mechanisms or after exposure to exogenous compounds (21, 22). DU is one of the external sources that invariably showed a number of indicators of oxidative stress such as elevation of ROS formation, induction of lipid peroxidation and GSH depletion (5, 9-11, 23). In order to determine if oxidative stress could be involved in DU cytotoxicity in fibroblast cells, we have attempted to elucidate the molecular events that occurred during U-induced oxidative stress in the human dermal fibroblast primary cells after exposure to different concentration of DU. 

The LDH leakage assay is based on release of the enzyme (14). In the present work the viability assays performed using LDH leakage assay test. It noted that in order to avoid overestimation or underestimation of the toxicity of a substance, incubations with various concentrations at many time points are required. Mortality that were observed after 24, 48 and 72 h incubation were 31, 52 and 82 percent respectively that we found a LC_50_ of 400 µM after 72 h incubation with DU. Also, as demonstrated in results, we confirmed that DU could induce oxidative stress in fibroblast cells when cells treatment with at least 250 µM DU. Among oxidative damage induced by ROS, the lipid membrane is a preferential target, causing lipid peroxidation which we showed that the MDA levels in fibroblast cells were increased as time and concentration dependent manner that again confirmed the role of ROS in DU toxicity and were parallel to cytotoxicity associated with uranyl acetate. The induction of lipid peroxidation by DU already has been reported in several studies on various cell types (9, 11).

It has been demonstrated that reductive activation of U (VI) by reduced CYP 2E1, or NADPH-P_450_ reductase and glutathione to U(V) and finally U (IV), provides enough redox cycling to generate considerable amounts of ROS. On the other hand, U(VI) can also cause mitochondrial membrane damage and significant collapse of mitochondrial membrane potential (12). So, mitochondria can be considered as a target for DU cytotoxicity, therefore we performed MTT assay test for evaluation of mitochondrial function. The present data showed that 24-72 h exposure of the human dermal fibroblast primary cells with DU significantly decline the mitochondrial functionality that showed using MTT dye reduction, which reveals the adverse impact of DU in mitochondrial function. 

The balance between free radical generation and the antioxidant systems is the key for maintaining a physiological intracellular redox status. Glutathione (GSH) is one of the main components of the cellular antioxidant systems that is critical to defense against reactive oxygen species (24). In this basis, we assayed the GSH content of the human dermal fibroblast primary cells exposed to different concentration of DU. As our data showed, GSH oxidation were significantly increased after 24, 48 and 72 h treatment with DU. Our results are in agreement with previous studies that showed glutathione oxidation was occurred as a consequence of DU induced-ROS formation (12). Generation of ROS has been widely viewed as being a common pathway to cell death from a variety of injurious agents (25), and apoptosis can be triggered by ROS (26, 27). Present work showed that the rate of apoptosis in fibroblast cells exposed to lower concentration of DU was significantly higher than the rate of necrosis. DU induced apparently more apoptotic death than necrotic death in fibroblast cells in low concentration but in longer incubation time and/or higher concentration, necrosis was the prominent mode of cell death in fibroblast cells. 

In conclusion, our results suggest that oxidative stress could be involved in cytotoxicity of DU in fibroblast cells. Glutathione and other antioxidants could protect the fibroblast cells against DU- induced cytotoxicity.

## References

[1] Bleise A, Danesi PR, Burkart W (2003). Properties, use and health effects of depleted uranium (DU): a general overview. J. Environ. Radioact.

[2] WHO (2001). Depleted Uranium: Sources, Exposure and Health Effects. Full Report.In WHO, in Department of Protection of the Human Environnment.

[3] U.S.EPA United States Environment Protection Agency 2002. http://www.epa.gov/superfund/resources/radiation/pdf/U.pdf.

[4] Sanchez DJ, Belles M, Albina ML, Sirvent JJ, Domingo JL (2001). Nephrotoxicity of Simultaneous Exposure to Mercury and Uranium in Comparison to Individual Effects of These Metals in Rats. Biol Trace Elem Res.

[5] Periyakaruppan A, Kumar F, Sarkar S, Sharma CS, Rames GT (2007). Uranium induces oxidative stress in lung epithelial cells. Arch Toxicol.

[6] Valko M, Izakovic M, Mazur M, Rhodes CJ, Telser J (2004). Role of oxygen radicals in DNA damage and cancer incidence. Mol. Cell. Biochem.

[7] Ibrahim D, Froberg B, Wolf A, Rusyniak DE (2006). Heavy metal poisoning: clinical presentations and pathophysiology. Clin. Lab.Med.

[8] Valko M, Rhodes CJ, Moncol J, Izakovic M, Mazur M (2006). Free radicals, metals and antioxidants in oxidative stress induced cancer. Chemico-Biol. Interact.

[9] Linares V, Bell´es M, Albina ML, Mayayo E, S´anchez DJ, Domingo JL (2005). Combined action of U and stress in the rat. II. Effects on male reproduction. Toxicol Lett.

[10] Taulan M, Paquet F, Maubert C, Delissen O, Demaille J, Romey MC (2004). Renal toxicogenomic response to chronic uranyl nitrate insult in mice. Environ. Health Perspect.

[11] Briner W, Murray J (2005). Effects of short-term and long-term depleted U exposure on open-field behavior and brain lipid oxidation in rats. Neurotoxicol. Teratol.

[12] Pourahmad J, Ghashang M, Ettehadi HA, Ghalandari R (2006). A search for cellular and milecular mechanisms involved in depleted uranium(DU) toxicity. Enviromental Toxicology.

[13] Taulan M, Paquet F, Maubert C, Delissen O, Demaille J, Romey MC (2004). Renal toxicogenomic response to chronic uranyl nitrate insult in mice. Environ Health Perspect.

[14] Decker T, Lohmann-Matthes ML (1988). A quick and simple method for the quantitation of lactate dehydrogenase release in measurements of cellular cytotoxicity and tumor necrosis factor (TNF) activity. J. Immunol. Methods.

[15] Reilly CA, Aust SD (1999). Measurement of Lipid Peroxidation.Current Protocols in Toxicology.

[16] Mosmann T (1983). Rapid colorimetric assay for cellular growth and survival :Application to proliferation and cytotoxicity assay. J. Immunol. Methods.

[17] Sadegh C, Schreck RP (2003). The Spectroscopic Determination of Aqueous Sulfite Using Ellman’s Reagent. MURJ.

[18] Tait JF, Smith C, Wood BL (1999). Measurement of Phosphatidylserine Exposure in Leukocytes and Platelets by Whole-Blood Flow Cytometry with Annexin V. Blood Cells, Molecules, and Diseases.

[19] Zhivotovsky B, Samali A, Orrenius S (1999). Assessment of Cell Toxicity.Current Protocols in Toxicology.

[20] Wang C, Sadovova N, Ali HK, Duhart HM, Fu X, Zou X, Patterson TA, Binienda ZK, Virmani A, Paule MG, jr WS, Ali ASF (2007). L-Carnitine Protects Neurons From 1-Methyl-4-Phenylpyridinium-Induced Neuronal Apoptosis In Rat Forebrain Culture. Neuroscience.

[21] Valko M, Morris H, Cronin MT (2005). Metals, toxicity and oxidative stress. Curr. Med. Chem.

[22] Pourahmad J, Hosseini MJ, Eskandari MR, Rahmani F (2012). Involvement of Four Different Intracellular Sites in Chloroacetaldehyde- Induced Oxidative Stress Cytotoxicity. Iranian J. Pharm. Res.

[23] Pourahmad J, Shaki F, Tanbakosazan F, Ghalandari R, Ettehadi HA, Dahaghin E (2011). Protective effects of fungal β-(1→3)-D-glucan against oxidative stress cytotoxicity induced by depleted uranium in isolated rat hepatocytes Hum. Exp. Toxicol..

[24] McCord JM (2000). The evolution of free radicals and oxidative stress. Am. J. Med.

[25] Ames BN, Shigenaga MK, Hagen TM (1993). Oxidants, antioxidants, and the degenerative diseases of aging. Proc. natn. Acad. Sci. U.S.A.

[26] Wood KA, Youle RI (1995). The role of free radicals , p53 in neuron apoptosis in vivo. J. Neurosci.

[27] Pourahmad J, Mortada Y, Eskandari MR, Shahraki J (2011). Involvement of Lysosomal Labilisation and Lysosomal/mitochondrial Cross-Talk in Diclofenac Induced Hepatotoxicity. Iranian J. Pharm. Res.

